# Adverse events after vaccination among HIV-positive persons, 1990–2016

**DOI:** 10.1371/journal.pone.0199229

**Published:** 2018-06-19

**Authors:** John R. Su, Carmen Ng, Paige W. Lewis, Maria V. Cano

**Affiliations:** Immunization Safety Office, Division of Healthcare Quality Promotion, National Center for Emerging and Zoonotic Infectious Diseases, Centers for Disease Control and Prevention, Atlanta, GA, United States of America; MCRI, AUSTRALIA

## Abstract

Human immunodeficiency virus (HIV) causes immune dysregulation, potentially affecting response to vaccines in infected persons. We investigated if unexpected adverse events (AEs) or unusual patterns of AEs after vaccination were reported among HIV-positive persons. We searched for domestic reports among HIV-positive persons to the Vaccine Adverse Event Reporting System (VAERS) during 1990–2016. We analyzed reports by age group (<19 and ≥19 years), sex, serious or non-serious status, live vaccine type (live versus inactivated), AEs reported, and CD4 counts. Of 532,235 reports received, 353 (0.07%) described HIV-positive persons, of whom 67% were aged ≥19 years, and 57% were male; most reports (75%) were non-serious. The most commonly reported inactivated vaccines were pneumococcal polysaccharide (27%) and inactivated influenza (27%); the mostly reported common live virus vaccines were combination measles, mumps, and rubella (8%) and varicella (6%). Injection site reactions were commonly reported (39%). Of 67 reports with CD4 counts available, 41 (61%) described persons immunocompromised at time of vaccination (CD4 count <500 cells/mm^3^), and differed from overall reports only in that varicella was the most common live virus vaccine (4 reports). Of 22 reports describing failure to protect against infection, 6 described persons immunocompromised at time of vaccination, among whom varicella vaccine was most common (3 reports). Of 66 reports describing live virus vaccines, 7 described persons with disseminated infection: 6 had disseminated varicella, 3 of whom had vaccine strain varicella-zoster virus. Of 18 reported deaths, 7 resulted from disseminated infection: 6 were among immunocompromised persons, 1 of whom had vaccine strain varicella-zoster virus. We identified no unexpected or unusual patterns of AEs among HIV-positive persons. These data reinforce current vaccine recommendations for this risk group. However, healthcare providers should know their HIV-positive patients’ immune status because immunocompromising conditions can potentially increase the risk of rare, but severe, AEs following vaccination with live virus vaccines.

## Introduction

Infection with Human immunodeficiency virus (HIV) remains a major public health concern: as of 2015, rates of new HIV diagnoses in the United States were 24.4 per 100,000 adolescent and adult men and 5.4 per 100,000 adolescent and adult women, [1] and the U.S. Centers for Disease Control and Prevention considers HIV infection a key Winnable Battle. [2] HIV infects CD4+ helper T-lymphocytes, depleting these cells and ultimately leading to acquired immunodeficiency syndrome (AIDS). Infection with HIV can lead to opportunistic infections, [3] and can also increase the risk of vaccine-preventable conditions like invasive pneumococcal infections and influenza-related complications. [4–6] For these reasons, recommendations for antibiotic prophylaxis and vaccines for HIV-positive persons exist. [7–10]

Even before progressing to AIDS, HIV infection leads to immune dysregulation. [11, 12] This dysregulation can affect response to and efficacy of vaccines in an HIV-positive person. Reduced antibody response to influenza, [13], hepatitis B, [14] and pneumococcal polysaccharide vaccines [15] have been observed among HIV-positive persons. Provided an HIV-person is not immunocompromised (i.e., CD4 <500 cells/mm^3^), live virus vaccines are still recommended if lack of immunity is documented. [7]

Given the immune dysregulation and modified response to vaccines that occur with HIV infection, the possibility exists of a different spectrum of adverse events (AEs) after vaccination among HIV-positive persons relative to HIV-negative persons. To explore this possibility, we reviewed domestic reports of AEs after vaccination among HIV-positive persons to the Vaccine Adverse Event Reporting System (VAERS) received during 1990 through 2016.

## Methods

### Data source

VAERS is a national spontaneous reporting system for monitoring AEs following US-licensed vaccines. [16] VAERS accepts reports from healthcare providers, vaccine manufacturers, vaccine recipients and others; signs and symptoms are coded using Medical Dictionary for Regulatory Activities (MedDRA) Preferred Terms (PTs). [17] MedDRA PTs are not medically confirmed diagnoses and a VAERS report may be assigned more than one MedDRA PT. Reports are classified as serious based on the Code of Federal Regulations if one or more of the following is reported: death, life-threatening illness, hospitalization or prolongation of existing hospitalization, or permanent disability. [18] Reported adverse health events might be clinically serious, but if they do not meet these regulatory criteria, they might not necessarily be classified as a serious report. Manufacturers are required to follow up serious reports (i.e., request and review medical records) prior to reporting to VAERS, so serious reports from manufacturers often do not include medical records with the VAERS report. For non-manufacturer serious reports, medical records are routinely requested and made available to VAERS personnel. However, because VAERS is a passive reporting system and dependent upon the reporter to provide clinical details and information, some data (e.g., clinical diagnoses) are not always available.

### Descriptive analysis

We searched the VAERS database for reports of AEs following vaccination among HIV-positive persons in the United States January 1, 1990 through December 31, 2016, with report received by February 28, 2017. We first searched for the PTs “blood HIV RNA”, “HIV antigen”, “HIV antigen positive”, “HIV infection”, “HIV test”, and “HIV test positive”. We conducted a second search for the text terms “HIV”, “HIV-positive”, “HIV positive”, HIV infection”, “HIV infected”, and “human immunodeficiency virus” in multiple fields for each report (e.g., symptom text, past medical history). We then combined the results of both searches, and deduplicated records, leaving only unique records for each report.

We reviewed all serious reports and all non-serious reports for which medical records were available; we also reviewed reported deaths. We stratified the data by age group (aged <19 years, and aged ≥19 years). [19, 20] For each age group, we analyzed reports by seriousness of report (serious, non-serious); sex; vaccines (live virus, inactivated), AEs; and when available, CD4 counts. We defined immunocompromised as a CD4 count of <500 cells/mm^3^ at time of vaccination. [21, 22] “Severely immunocompromised” is defined as a CD4 count of <200 cells/mm^3^, and is the usual threshold to determine if an HIV-positive person should receive a live virus vaccine; [7] for children aged less than 6 years, higher CD4 count thresholds can apply. [23, 24] Our intent was to describe both persons who were severely immunocompromised, and immunocompromised persons with a CD4 count >200 cells/mm^3^.

## Results

During January 1, 1990 through December 31, 2016, VAERS received 532,235 reports. Of these reports, we identified 353 (0.07%) describing persons who were HIV-positive (Table 1); medical records were available for 48 reports (14%). Most (266, 75%) reports were non-serious. Among reports that included age, most (86%) were among persons aged ≥19 years. Among reports with data on sex available, most (68%) were males. The most common vaccine types in the reports were inactivated influenza (IIV) (96, 27%) and pneumococcal polysaccharide (PPSV23) (94, 27%). Combined measles, mumps, and rubella (MMR) was the most common live virus vaccine (27, 8%). The most common AEs were injection site reactions (138, 39%), pain (92, 26%), and fever (63, 18%).

**Table 1:** Characteristics of reports to VAERS among HIV-positive persons, 1990–2016.

	Age group			Overall
	<19 years (n = 39) (%)	19 years and older (n = 236) (%)	Unknown (n = 78) (%)	N = 353 (%)
**Seriousness**				
Non-serious	22 (56)	175 (74)	69 (88)	266 (75)
Serious, non-death	10 (26)	52 (22)	7 (9)	69 (20)
Death	7 (18)	9 (4)	2 (3)	18 (5)
**Sex**				
Male	21 (54)	160 (68)	19 (24)	200 (57)
Female	17 (44)	72 (31)	5 (6)	94 (27)
Unknown	1 (3)	4 (2)	54 (69)	59 (17)
Vaccines, live*				
MMR	6 (15)	13 (6)	9 (12)	28 (8)
Varicella	12 (31)	8 (3)	1 (1)	21 (6)
Zoster	0 (0)	9 (4)	2 (3)	11 (3)
LAIV	0 (0)	7 (3)	0 (0)	7 (2)
Yellow fever	0 (0)	2 (1)	0 (0)	2 (1)
Smallpox/Vaccinia	0 (0)	2 (1)	0 (0)	2 (1)
OPV	2 (5)	0 (0)	0 (0)	2 (1)
Vaccines, inactivated*				
Influenza, inactivated	1 (3)	86 (36)	9 (12)	96 (27)
PPSV23	6 (15)	69 (29)	19 (24)	94 (27)
Hepatitis^a^	4 (10)	46 (19)	33 (42)	83 (24)
PNC	9 (23)	16 (7)	4 (5)	29 (8)
Tdap or DTaP^†^	9 (23)	17 (7)	1 (1)	27 (8)
HPV	3 (8)	10 (4)	4 (5)	17 (5)
Adverse events**				
Injection site reaction ^b^	23 (59)	110 (47)	5 (6)	138 (39)
Pain	4 (10)	86 (36)	2 (3)	92 (26)
Fever	6 (15)	53 (22)	4 (5)	63 (18)
Rash	6 (15)	42 (18)	0 (0)	48 (14)
Headache	3 (8)	27 (11)	1 (1)	31 (9)
Redness	2 (5)	24 (10)	3 (4)	29 (8)
Drug ineffective^††^	5 (13)	9 (4)	8 (10)	22 (6)
Muscle pain	1 (3)	18 (8)	0 (0)	19 (5)
Nausea	1 (3)	17 (7)	0 (0)	18 (5)
Pneumonia	3 (8)	10 (4)	4 (5)	17 (5)
Infection	5 (13)	10 (4)	0 (0)	15 (4)

* Includes both vaccines administered singly, and concomitantly with other vaccines; MMR = combination measles, mumps, rubella vaccine; LAIV = live attenuated influenza vaccine; OPV = oral polio vaccine; PPSV23 = pneumococcal polysaccharide vaccine; PNC = pneumococcal conjugate vaccine.

^†^ Includes DT (combination diphtheria and tetanus toxoids), DTP (combination DT and whole cell pertussis.

vaccine) and combination vaccines that include DTaP (combination DT and acellular pertussis vaccine) and Tdap (combination tetanus and diphtheria toxoids and acellular pertussis vaccine).

^a^ Includes hepatitis A, hepatitis B, and combination hepatitis A and B vaccines.

** Because more than one adverse event can be reported per report, the sum of percentages can exceed 100%.

^††^ Includes infection with agent against which vaccine should protect.

^b^ Includes cellulitis at injection site.

Among reports in persons aged <19 years, median age was 5 years (range 0 to 17 years), with 75% of persons aged <11 years. The most common vaccines in reports were varicella (12, 31%), pneumococcal conjugate (9, 23%), and combinations of diphtheria and tetanus toxoids and pertussis (whole cell and acellular) vaccines (9, 23%). Among persons aged ≥19 years, the most common vaccines were IIV (86, 36%) and PPSV23 (69, 29%). Regardless of age group, the most commonly reported AEs were injection site reactions.

CD4 counts were available for 67 reports, of which 41 described persons who were immunocompromised (CD4 <500 cells/mm^3^) (Table 2). Most (38, 93%) of these 41 persons were immunocompromised at time of vaccination, were aged ≥19 years (33, 80%), and male (29, 71%). Regardless of CD4 count, comparable proportions of reports were serious (65% for CD4 ≥500 and 66% for CD4 <500 cells/mm^3^). Varicella was the most common live virus vaccine (5, 7%), and IIV (27, 40%) and PPSV23 (19, 28%) were the most common inactivated vaccines; injection site reactions and pain were the most commonly reported AEs. Of the 41 immunocompromised persons, 24 (59%) had CD4 ≥200 cells/mm^3^: 1 (4%) was aged <19 years, and 3 (13%) received live virus vaccines. Of 17 persons with CD4 <200 cells/mm^3^, 4 (24%) were aged <19 years, and 8 (47%) received live virus vaccines. Persons with CD4 counts ≥200 and <200 cells/mm^3^ were otherwise comparable by seriousness of report, sex, and reported AEs.

**Table 2:** Adverse events reported to VAERS among HIV-positive persons by CD4 count, 1990–2016.

	CD4 count (cells/mm^3^)		
	<500 (n = 41) (%)	≥500 (n = 26) (%)	Total (n = 67) (%)
**Seriousness**			
Non-serious	14 (34)	9 (35)	23 (34)
Serious, non-death	20 (49)	14 (54)	34 (51)
Death	7 (17)	3 (12)	10 (15)
**Sex**			
Male	29 (71)	14 (54)	43 (64)
Female	9 (22)	11 (42)	20 (30)
Unknown	3 (7)	1 (4)	4 (6)
Vaccines, live*			
Varicella	4 (10)	1 (4)	5 (7)
MMR	1 (2)	1 (4)	2 (3)
Zoster	2 (5)	0 (0)	2 (3)
Yellow fever	2 (5)	0 (0)	2 (3)
Smallpox/Vaccinia	2 (5)	0 (0)	2 (3)
LAIV	0 (0)	1 (4)	1 (1)
OPV	0 (0)	0 (0)	0 (0)
Vaccines, inactivated*			
Influenza, inactivated	13 (32)	14 (54)	27 (40)
PPSV23	13 (32)	6 (23)	19 (28)
PNC	2 (5)	4 (15)	6 (9)
Hepatitis^a^	3 (7)	2 (8)	5 (7)
Tdap or DTaP^†^	3 (7)	2 (8)	5 (7)
HPV	1 (2)	0 (0)	1 (1)
Adverse events**			
Injection site reaction ^b^	16 (39)	24 (92)	40 (60)
Pain	27 (66)	8 (31)	35 (52)
Fever	13 (32)	5 (19)	18 (27)
Headache	9 (22)	4 (15)	13 (19)
Rash	8 (20)	5 (19)	13 (19)
Chills	9 (22)	1 (4)	10 (15)
Nausea	7 (17)	2 (8)	9 (13)
Paresthesia	3 (7)	6 (23)	9 (13)
Muscle weakness	3 (7)	5 (19)	8 (12)
Drug ineffective^††^	6 (15)	2 (8)	8 (12)

* Includes both vaccines administered singly, and concomitantly with other vaccines; MMR = combination measles, mumps, rubella vaccine; LAIV = live attenuated influenza vaccine; OPV = oral polio vaccine; PPSV23 = pneumococcal polysaccharide vaccine; PNC = pneumococcal conjugate vaccine.

^†^ Includes DT (combination diphtheria and tetanus toxoids), DTP (combination DT and whole cell pertussis vaccine) and combination vaccines that include DTaP (combination DT and acellular pertussis vaccine).

^a^ Includes hepatitis A, hepatitis B, and combination hepatitis A and B vaccines.

** Because more than one adverse event can be reported per report, the sum of percentages can exceed 100%.

^††^ Includes infection with agent against which vaccine should protect.

^b^ Includes cellulitis at injection site.

Twenty-two (6%) of the 353 reports included the MedDRA PT “drug ineffectiveness” (e.g., vaccine failure): 10 reports were serious, including 4 deaths (Table 3). Notably, reports for hepatitis vaccine described failure to seroconvert, while reports for PPSV23 and *Haemophilus influenzae b* vaccines described sepsis. Reports for MMR and varicella vaccines described infection with measles or varicella zoster virus (VZV) post-vaccine. CD4 counts were available for 8 of these 22 reports: 6 (75%) of these 8 reports described immunocompromised persons.

**Table 3:** Reports to VAERS of drug ineffectiveness/vaccine inefficacy by immune status among HIV-positive persons, 1990–2016.

	Immuno-compromised* (n = 6)	Not immuno-compromised* (n = 2)	Not reported (n = 14)	Total (n = 22)
**Seriousness**				
Non-serious	1	0	11	12
Non-death, serious	2	1	3	6
Death	3	1	0	4
**Age**				
<19 years	3	0	2	5
≥19 years	3	2	4	9
Not reported	—	—	8	8
**Sex**				
Male	4	1	7	12
Female	2	1	1	4
Unknown	—	—	6	6
Vaccines, live**				
Varicella	3	1	1	5
MMR	1	0	1	2
Vaccines, inactivated**				
Hepatitis^a^	0	0	10	10
PPSV23	1	1	2	4
Tdap or DTaP^†^	1	0	0	1
HIBV	0	0	1	1

* “Immunocompromised” is CD4<500 cells/mm^3^; “Not immunocompromised” is CD4 ≥500 cells/mm^3^.

** Includes both vaccines administered singly, and concomitantly with other vaccines; MMR = combination measles, mumps, rubella vaccine; PPSV23 = pneumococcal polysaccharide vaccine; HIBV = *Haemophilus influenzae b* vaccine.

^†^ Includes DT, DTP (combination DT and whole cell pertussis vaccine) and combination vaccines that include DTaP.

^a^ Includes hepatitis A, hepatitis B, and combination hepatitis A and B vaccines.

Of the 66 reports involving live virus vaccines, MMR and varicella were most common (Table 1); 14 (21%) described infection with measles or VZV post-vaccination, including 7 disseminated infections. Six persons with disseminated infections had disseminated varicella (CD4 of <1, <4, 8, 15 cells/mm^3^, and unreported (2 persons)), including a woman aged 29 years with VZV retinal necrosis (unknown if vaccine strain) (CD4 = 15 cells/mm^3^); 1 person had wild-type measles encephalitis (CD4 = 300 cells/mm^3^). Three persons with disseminated varicella had vaccine strain VZV, including a woman aged 15 years with cerebellar lesions and VZV (+) cerebrospinal fluid.

We identified 18 reports of death: 7 (41%) were among persons aged <19 years, 9 (53%) among persons aged ≥ 19 years, and 2 without age reported; 13 were males, 4 were female, and 1 was without sex reported; 10 (59%) had known CD4 counts, 7 of whom were immunocompromised (i.e., <500 cells/mm^3^). Among these 7 immunocompromised persons, 6 deaths were from disseminated infection: 2 from pneumococcal pneumonia, 1 from pneumonia (cause unspecified), 1 from wild-type measles encephalitis, and 2 from disseminated varicella. Of these deaths, only 1 involved vaccine strain virus: a female aged 15 years died from disseminated varicella after receiving varicella vaccine, who had vaccine-strain VZV in her cerebrospinal fluid. Additionally, 1 person died from an acute cerebrovascular accident. Of the 11 persons not known to be immunocompromised, causes of death were acute hemorrhagic pancreatitis, cardiomegaly, cerebral infarction, measles pneumonitis (not known if vaccine-strain), metastatic esophageal carcinoma, multiorgan failure, pneumococcal pneumonia, and *Pneumocystis* pneumonia; cause of death was not reported for 3 cases. Only 1 person not known to be immunocompromised died from disseminated infection: a male aged 48 years who died after PPVS23 from pneumococcal pneumonia and sepsis.

## Discussion

Our review of AEs reported to VAERS among HIV-positive persons revealed no unexpected AEs or unusual patterns of AEs: reported AEs were consistent with known health consequences of HIV infection and immunocompromised immune status, including one death involving disseminated varicella in a severely immunocompromised patient who had vaccine-strain VZV. Severe immunodeficiency is a contraindication to vaccination with live virus vaccines. [7] Our findings reinforce current general recommendations that for HIV-positive persons, inactivated vaccines are indicated, and live attenuated vaccines can be considered if severe immunodeficiency (e.g., CD4 <200 cells/mm^3^ among persons aged 6 years and older [23, 24]) and physical signs of such immunodeficiency are absent; [7, 8] providers might consider obtaining CD4 counts prior to administering live virus vaccines for patients known to be HIV-positive. If a patient is at risk for HIV infection (e.g., HIV-positive birth mother, known risk population), the provider might consider screening for HIV [25] prior to administering live virus vaccines.

The predominance of reports among males (and among persons aged ≥ 19 years) likely reflects the epidemiology of HIV in the United States, with most HIV infection acquired by sexual contact, often among males. [1] In contrast, the comparable number of reports among males and females aged <19 years likely reflects non-sexual transmission of HIV: 75% of such persons were aged <11 years (e.g., before sexual activity and intercourse would be expected).

The high proportions of reports involving live virus vaccines among HIV-positive persons aged <19 years (relative to ≥19 years), and of inactivated vaccines among persons aged ≥19 years, reflect the vaccination schedules for persons in their respective age groups. [26, 27] Regardless of age group, the data revealed no new or unexpected patterns of AEs (Table 1) among HIV-positive persons; the reported AEs have been previously described for these vaccines. [28]

The reports of “drug ineffective” (in this case, failure of the vaccine to protect against infection) among HIV-positive persons with CD4 <500 cells/mm^3^ (Table 2) reflect the immunocompromised status of these persons. Previous publications describe disseminated varicella in HIV-positive persons, including retinal necrosis and other central nervous involvement similar to the conditions described in our analysis. [29–31] Disseminated varicella after administration of varicella vaccine has also been described. [32, 33] Antibody responses to PPSV23 can be lower in immunocompromised HIV-positive persons, [15, 34, 35] and this lower response has been associated with an increased risk of invasive pneumococcal disease. [36, 37] Such impaired antibody response and poorer clinical outcomes have also been observed with IIV among immunocompromised HIV-positive persons. [6, 38, 39] These observations reinforce current recommendations of avoiding live virus vaccines in severely immunocompromised persons, [7, 40, 41] and are reminders that inactivated vaccines can be less effective in immunocompromised persons, like HIV-positive persons.

VAERS data are subject to the limitations of passive surveillance in general, including under-reporting, reporting biases, inconsistent data quality and completeness (e.g., not all reports have medical records available for review), and secular changes in reporting over time. [16] Also, we lack an unvaccinated, HIV-negative comparison group, and we lack denominator data (e.g., doses of vaccine administered), which would allow calculation of gross rates of AEs after vaccination. For these and other reasons, we generally cannot determine if a vaccine caused an AE using VAERS data alone. An exception might be the identification of vaccine strain VZV in cases of disseminated varicella post-vaccination, which demonstrates unequivocal laboratory evidence. Despite these limitations, VAERS can detect unusual or unexpected patterns of reported AEs, [42, 43] including among HIV-positive persons, and can help identify possible vaccine safety concerns requiring further investigation. The current analysis detected no such concerns.

Vaccine preventable diseases carry an increased risk for poor outcomes among HIV-positive persons, for whom vaccines are a key preventive measure. The data in this analysis are reassuring, finding no unusual AEs or vaccine safety concerns among HIV-positive persons. Health care providers should vaccinate their HIV-positive patients as recommended by current guidelines; [8, 26, 27] the recent recommendation that inactivated herpes zoster (Shingles) vaccine be used in preference to the live virus varicella vaccine is particularly pertinent to HIV-positive persons. [44] Providers should be aware that live virus vaccines are contraindicated in severely immunocompromised persons, and should therefore be aware of their patient’s immune status at time of vaccination (e.g., CD4 count), because of the risk of rare, but severe outcomes.
